# In-Situ Surface Modification of Terpinen-4-ol Plasma Polymers for Increased Antibacterial Activity

**DOI:** 10.3390/ma13030586

**Published:** 2020-01-27

**Authors:** Avishek Kumar, Ahmed Al-Jumaili, Kateryna Bazaka, Peter Mulvey, Jeffrey Warner, Mohan V. Jacob

**Affiliations:** 1 Electronics Materials Lab, College of Science and Engineering, James Cook University, Townsville 4811, Australia; Avishek.Kumar@my.jcu.edu.au (A.K.); Ahmed.Aljumaili@my.jcu.edu.au (A.A.-J.); Katia.Bazaka@anu.edu.au (K.B.); 2 Institute for Future Environments, Queensland University of Technology, Brisbane 4000, Australia; 3 Research School of Electrical, Energy and Materials Engineering, The Australian National University, Canberra 2601, Australia; 4 AITHM, Immunology & Infectious Disease, Australian Institute of Tropical Health & Medicine, James Cook University, Townsville 4811, Australia; Peter.Mulvey@jcu.edu.au; 5 Discipline of Biomedicine, College of Public Health, Medical and Veterinary Sciences, James Cook University, Townsville 4811, Australia; Jeffrey.Warner@jcu.edu.au

**Keywords:** in-situ polymer surface modification, ZnO functionalization, plasma polymers, antibacterial coatings

## Abstract

Surface modification of thin films is often performed to enhance their properties. In this work, in situ modification of Terpinen-4-ol (T4) plasma polymer is carried out via simultaneous surface functionalization and nanoparticle immobilization. Terpinen-4-ol plasma polymers surface were decorated with a layer of ZnO nanoparticles in an oxygen plasma environment immediately after polymer deposition. A combination of hydrophilic modification and ZnO nanoparticle functionalization of the T4 polymer surface led to an enhancement in antibacterial properties by factor of 3 (from 0.75 to 0.25 CFU.mm^−2^). In addition, ZnO nanoparticle-modified coatings demonstrated improved UV absorbing characteristics in the region of 300–400 nm by 60% relative to unmodified coatings. The ZnO modified coatings were transparent in the visible region of 400–700 nm. The finding points towards the potential use of ZnO nanoparticle-modified T4 plasma polymers as optically transparent UV absorbing coatings.

## 1. Introduction

Bacterial adhesion to material surface is the primary step in biofilm development. Biofilm formation can potentially have severe health and industrial repercussions [1,2], with examples including implant-associated infections [3,4] and marine fouling of environmental sensors, pontoons [5,6,7]. Planktonic cells of many microbial species have a tendency to form a biofilm matrix on surfaces as a means of protection against hostile environment and predation. Once the biofilm enters the tertiary phase of development, killing of biofilm-residing cells, and biofilm dissolution and removal become challenging. According to some estimates, between 2001 and 2007, hospital acquired infections (HAI) have cost US hospitals $40 billion yearly [8].

Surface modification of materials has been frequently used to minimize microbial adhesion [9,10]. Surfaces functionalized with enzymes, antibiotics and biocides have also been used in the effort to combat microbial attachment [11,12,13]. Hydrophilic/hydrophobic modification and immobilization of nanoparticles (Nps), such as Cu, ZnO, and Ag, onto the surface of material have been used separately as an effective strategy to reduce biofilm formation [14,15,16,17,18]. Surfaces modified to display hydrophilic behavior have shown superior antifouling properties [19,20,21]. Hydrophilic surfaces form hydrogen bonds with water molecules, leading to the formation of a hydration layer at the water/surface interface. This hydration layer acts as an energetic barrier and prevents the adhesion of fouling molecules. For example, Wu et al. used atom transfer radical polymerization to impart hydrophilicity on silicon surfaces using N-vinylpyrrolidone (PVP). The Si-PVP surfaces were characterized by a low water contact angle of 24° and showed reduced adsorption of human serum albumin, lysosome and fibrinogen by 93, 81 and 71%, respectively [22]. Poly (ethylene glycol) (PEG) has been widely used for hydrophilic modification of surfaces for antifouling applications. PEG functionalized surfaces have been widely used for the development of safer central venous catheters [21], hemodialysis membranes [23], and biosensors and biochips [24,25].

Surface modification techniques such as block co-polymerization [26,27], covalent grafting of hydrophilic groups to surfaces [28], nanoparticle functionalization [29,30], atom transfer radical polymerization [31], Radio Frequency plasma deposition and modifications of surfaces have been used to fabricate surfaces showing antifouling properties. Kim et al. modified nanofiltration thin film composite membranes to be more hydrophilic by a treatment with NH_3_ plasma. The membranes showed superior antifouling properties on account of their increased hydrophilicity [32].

Nanoparticles have found wide bio application on account of, among many other attributes, their broad spectrum antibacterial activity [33,34]. Among a wide range of particles, zinc oxide nanoparticles have demonstrated enhanced antibacterial and antifouling properties [35,36]. Incorporation of ZnO nanoparticles into a polymer matrix such as that of polyvinylidene fluoride (PVDF) [37], polysulfide (PSF) [38] and polyethersulfone (PES) [39] enhanced the antifouling activity of these polymer matrices when used as ultrafiltration membranes. Chitosan-ZnO nanocomposites have demonstrated an effective marine antifouling activity against *Pseudoalteromonas nigrifaciens* bacteria and the diatom *Navicula sp*. [40].

Terpinen-4-ol plasma polymers are a recent entry in the range of biodegradable polymer materials that show promising antimicrobial activity and have both biomedical and marine applications [7,41,42,43]. This study investigates the effect of surface modification of terpinen-4-ol plasma polymers via in-situ hydrophilic modification via oxygen plasma treatment and ZnO nanoparticle (Np) immobilization on antibacterial activity against *Escherichia coli*. Typically, plasma enhanced chemical vapor deposition (PECVD) of ZnO films requires high processing temperatures [44]. However, Terpinen-4-ol plasma polymers will lose their antimicrobial property if deposited at a high temperature due to excessive loss of original chemical functionality of the monomer. The approach used in this study overcomes this challenge by proposing a method by which the desired antimicrobial property of terpinen-4-ol can be retained and further enhanced by ZnO Np immobilization and surface wettability modification of the polymer.

## 2. Film Fabrication and Characterization

### 2.1. Materials

Terpinen-4-ol (C_10_H_18_O, Mw = 154.24 g/mol, Purity > 99%) and zinc acetylacetonate (Zn(acac)_2_) were purchased from (Australian Botanical Products, Hallam, Australia) and (Sigma Aldrich, St.Louis, MO, USA), respectively, and used without any further modification. Microscope glass cover slips (Φ = 19 mm, ProSciTech, Kirwan, Australia) and silicon wafers (University Wafer, Boston, MA, USA) were used as substrates onto which the polymer was deposited. The substrates were cleaned in ultrasonic bath for 30 min in 5% solution of Decon 90 (Decon Laboratories Limited, King of Prussia, PA, USA), followed by double distilled water, acetone and propanol. High purity oxygen and argon gas (purity > 99.9%) were procured from BOC Gas, Townsville, Australia.

### 2.2. Thin Film Fabrication

**Figure 1:**
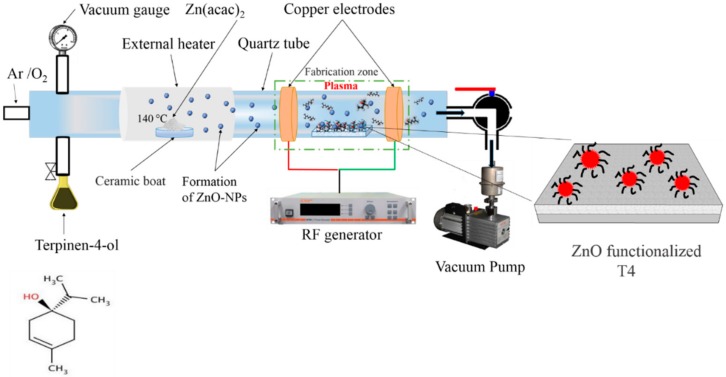
Schematic of plasma polymerization apparatus setup.

ZnO nanoparticle-functionalized terpinen-4-ol films were fabricated in a custom built capacitively coupled tubular (Quartz, l = 80 cm, d = 5 cm) radio frequency reactor (13.56 MHz, Advanced Energy, Fort Collins, CO, USA). Electrode separation was maintained at 8 cm for all the depositions. Substrates were placed 1 cm away from the leading electrode. Zinc acetyl acetonate hydrate [Zn(C_5_H_7_O_2_)_2_ xH_2_O] was placed in the clean ceramic boat and loaded in the middle of the reactor. External heating was used to vaporize Zn(acac)_2_ at a temperature of 140 °C. No external substrate heating was used during the deposition. A schematic of plasma polymerization apparatus and chemical structure of monomer is shown in Figure 1. The substrate temperature was found to be 40 °C during ZnO Np immobilization onto terpinen-4-ol surfaces. Terpinen-4-ol monomer flow rate was kept constant at 29 cm^3^/min. The polymerization of terpinen-4-ol was carried out at 10 W and the process pressure of 5 × 10^−2^ mbar. Terpinen-4-ol plasma polymers deposited at power of 10 W have been found to retain their inherent antimicrobial properties. Films deposited at higher power than 10 W have shown no antimicrobial properties, whereas one deposited at lower power are unstable under aqueous conditions [7,45]. A fine-tuning of plasma power, process pressure, and flow rate have been found to deposit a Terpinen-4-ol plasma polymer retaining a maximum antimicrobial property of the monomer. Either argon or oxygen were used as a carrier gas with latter providing hydrophilic modification of the surface of the as-deposited terpinen4-ol polymer matrix. Deposition of T4 monomer alone in either oxygen or argon plasma led to the wettability modification imparting hydrophilic/hydrophobic character to T4 polymer as compared to T4 polymer deposited in absence of carrier gas. Passing Zn(acac)_2_ vapor over the freshly deposited polymer led to in situ functionalization of the surface with ZnO nanoparticles. Flow rate of the argon and oxygen carrier gas was kept constant at 6 cm^3^/min. The desired thickness of the polymer was achieved by adjusting the deposition time.

### 2.3. Thin Film Characterization

Film thickness was estimated by spectroscopic ellipsometry (VASE J. A. Woollam, M2000 D, Lincoln, NE, USA). Measurements were performed in the wavelength range of 200–1000 nm at three different angle of incidence (55°, 60° and 65°). The film thickness were modelled using Cauchy function. The morphology of the films was characterized by field emission scanning electron microscopy at 10 KV (FE-SEM, Hitachi SU 500, Tokyo, Japan). The chemical composition of the films was estimated by FT-IR spectroscopy (PerkinElmer, Waltham, MA, USA; Spectrum 100, Staten Island, NY, USA) in Attenuated Total Reflectance (ATR) mode. Films were deposited on Potassium bromide (KBR) pellets for ATR-FTIR characterization and spectra were obtained at resolution of 4 cm^−1^ averaged over 124 scans. All the acquired FT-IR data were thickness normalized. Hydrophilic/hydrophobic character of the films was determined by static contact angle measurement by means of a goniometer (KSV CAM 101, KSV instruments, Helsinki, Finland) using three liquids (Water, Ethane diol, Di-iodomethane). Five measurements were performed per sample averaged over five samples using a drop volume of 3 μL. The Young–Laplace fitting and Van–Oss–Chaudhury–Good method was used to get contact angle and surface energy values, respectively. Raman spectra of the films were acquired with a confocal Raman microscope (Witec Alpha 300 Access, WITec GmBH, Ulm, Germany). The Raman spectra were recorded in the range of 100–4000 cm^−1^. Surface roughness parameters were examined using atomic force microscopy in a tapping mode using Atomic force microscopy (NT-MDT Spectrum Instruments, Zelenograd Moscow, Russian Federation). The average roughness was measured for 3 μm × 3 μm image collected at room temperature.

### 2.4. Bacterial Assay

Antibacterial activity of films was evaluated against *E. coli* (ATCC-924). *E. coli* cells were cultured in Luria–Betani (LB) broth at 37 °C for 24 h to reach the log phase. The bacterial solution was diluted to 5 × 10^5^ colony forming units (CFU)/mL. The coated and uncoated glass substrates (control) were sterilized by subsequent washing in ethanol and phosphate-buffered saline (PBS) solution. The sterilized films and controls were placed into 12-well cell culture plates (Falcon Laboratories, Colorado Springs, CO, USA). In addition, 2 mL of *E. coli* suspension was placed onto samples into each well and incubated at 37 °C for 24 h. Each sample was gently washed once with 10 mL of sterile PBS [46] and placed into 50 mL polyethylene tubes containing 5 mL of sterile PBS solution. Tubes were sonicated for 5 min to detach bacterial cells. Furthermore, 10 µL of bacterial suspension was added to 90 µL of prewarmed LB broth. In addition, 100 µL of the diluted bacterial suspension was plated onto nutrient agar. Agar plates were brooded at 37 °C for 24 h, and the number of CFU were counted. All tests were carried out with three replicates.

## 3. Results and Discussion

### 3.1. Deposition Rate

**Figure 2:**
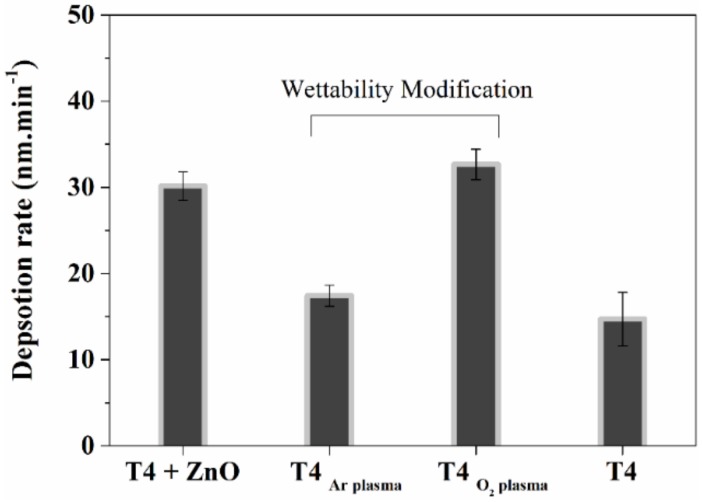
Deposition rate of T4 on silicon wafer substrate during wettability and nanoparticle modification. Deposition in either argon or oxygen plasma led to wettability modification. ZnO nanoparticle functionalization of T4 polymers is carried in O_2_ plasma.

Wettability and ZnO modification of T4 was carried out via plasma enhanced chemical vapor depositions (PECVD). Deposition rate of 17.43, 32.67 nm min^−1^ was found for T4 plasma polymers deposited using Argon and Oxygen as carrier gas, respectively. Deposition rate of T4 in the absence of any carrier gas and during in-situ ZnO functionalization was 14.7 and 30.15 nm min^−1^, respectively. The thickness of prepared plasma polymer film was kept constant at 300 nm by varying deposition time. Silicon wafers were used as substrates during deposition. Deposition rates (R_d_) of coatings are shown in Figure 2. The deposition rate of the T4 in the absence of carrier gas is also shown for comparison. As seen in Figure 2, R_d_ in oxygen plasma is higher than in the Ar plasma. The higher effective flow rate (F = F _m_ + aF_c_) of gases (O_2_ + T4 monomer) in oxygen plasma contributes to higher R_d_. Formation of oxygen radicals and their reaction with film-forming species enhance the deposition rate. In addition, the correction flow factor *a* in the above equation is 0.6 for the oxygen and (0.05–0.1) for argon [47]. Argon when used as a carrier gas in PECVD has been found to increase the monomer fragmentation [48]; however, the deposition mechanism remains unaffected [49].

### 3.2. Film Composition

**Figure 3:**
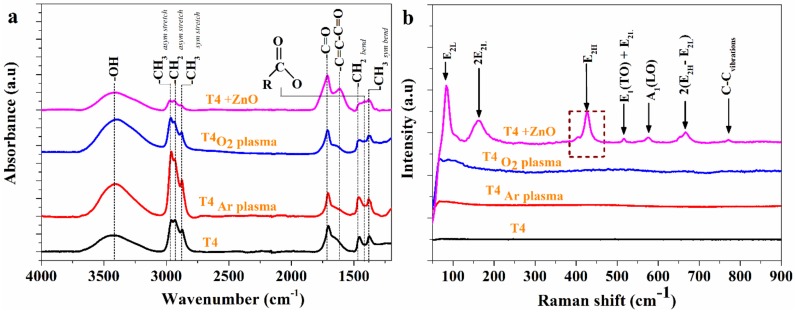
(**a**) ATR-FTIR spectra of ZnO nanoparticle functionalized and wettability modified T4 surfaces; (**b**) Raman spectra of modified T4 surfaces.

Figure 3a shows the Attenuated total reflection-Fourier-transform infrared spectroscopy (ATR-FTIR) spectra of all modified T4 films. The FTIR spectrum of T4-ZnO nanocomposite shows the characteristic peaks at 1590, 1521, 1448, 1390 and 1254 cm^−1^. The peaks occurring at these positions are characteristic of pp-Zn(acac)_2_ [50]. The appearance of the band at 1710 cm^−1^ in all the deposited films is indicative of the formation of −C=O group during deposition, irrespective of the surface modification. The T4_O2 plasma_ and T4_Ar plasma_ films show the characteristic peaks at 2800–3000 cm^−1^ (aliphatic C-H stretching) and 3400 cm^−1^ (O-H stretching vibration). The intensity of the above peaks (2800–3000 cm^−1^) is greatly reduced in the case of T4-ZnO nanocomposites. It is interesting to note that there is the formation of carboxylate and conjugated ketone functionalities during ZnO modification of T4 in O_2_ plasma. Attachment of acetylacetonates groups from Zn(acac)_2_ on T4 explains their occurrence.

Raman spectroscopic studies of the samples was done to get a qualitative estimate of ZnO nanoparticles in the deposited films. Figure 3b represents Raman spectra of ZnO functionalized T4 films. Spectra of T4 deposited in the absence and presence of carrier gas (either O_2_ or Ar) are also shown for comparison. Wurtzite ZnO is characterized by a set of eight optical phonons: Γ_opt_ = A_1_ + E_1_ + 2E_2_ + 2B_1_. A_1_ and E_1_ are polar, whereas and E_2_ modes are non-polar and Raman active [51,52]. The E_2H_ vibration mode at 440 cm^−1^ is representative of wurtzite phase of ZnO. The E_2H_ phonon frequency is red shifted by 12 cm^−1^ in the film (T4 + ZnO). Defect formation [53] on nanoparticles and phonon confinement [54] gives rise to this peak shift. The other peaks at about 83, 400 and 574 cm^−1^ are representative of E_2L_, A_1_ (TO) and A_1_ (LO) fundamental phonon mode of wurtzite ZnO. The other peaks at 514, 666 and 766 cm^−1^ are assigned to E_1_ (TO) + E_2L_, 2 (E_2H_ − E_2L_) and A_1_ (TO) + E_2L_ multiphoton scattering modes. Most of the Raman peaks of deposited films have been found to be red shifted as compared to the well-established ones in the literature [55]. The bonding of organic T4 and acetate molecules on ZnO surface is the possible reason for this lowering of vibrational frequency [56,57].

The thermal decomposition of zinc acetylacetonate hydrate [Zn(C_5_H_7_O_2_)_2_.xH_2_O] has been found to yield metallic zinc and zinc oxide [30,58,59]. The ZnO can get incorporated into polymer surface as metallic zinc or zinc oxide. At temperature above 110 °C, zinc acetylacetonate hydrate has been found to go through the complex process of decomposition. Initially, there is single step dehydration followed by the phase transition, fusion evaporation and decomposition of anhydrous zinc acetylacetonate [60]. There is a tendency for ZnO and Zn particles to segregate in the polymer surface as they have higher cohesive energy than the polymers. When metals are incorporated into the polymer surface, they have been found to go through the various processes. The processes such as desorption, diffusion into bulk or random rolling on the surface has been found to occur [60].There is very weak interaction between nanoparticles and polymer surfaces.

### 3.3. Coating Wettability

**Figure 4:**
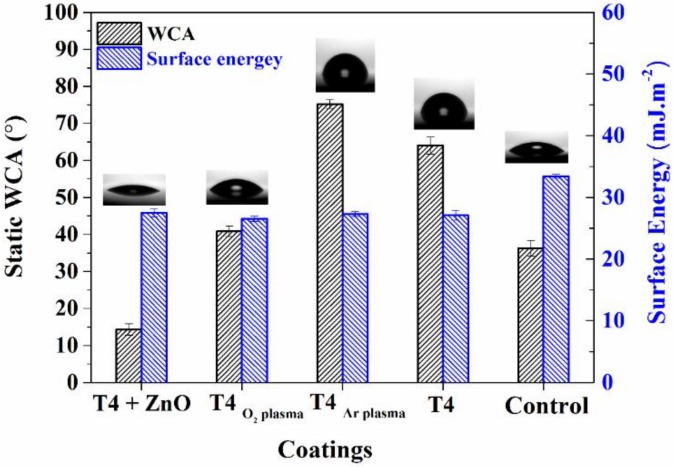
Static water contact angle and surface energy of modified T4 coatings. The inset image shows the contact angle less than 90°. Control consists of glass coverslip.

Water contact angle (WCA) measurements provide information about the hydrophilic/hydrophobic nature of the films. Figure 4 shows the WCA measurements on the modified T4 films along with the surface energy. Table 1 lists contact angles for three liquids on the modified surfaces used for free energy calculations. The contact angles on the surfaces of T4 and control samples are also shown for comparison. It can be observed that deposition in oxygen plasma results in the film with hydrophilic attributes. The ZnO-modified T4 shows highly hydrophilic character (WCA < 15°). Polar character of ZnO and deposition in oxygen plasma render these films highly hydrophilic. T4 films deposited in argon and in oxygen plasmas exhibited contact angles of 75° and 40°, respectively. The high proportion of hydrophobic carbonaceous −CH_x_ functionalities evident in FTIR spectra explains the increase in the contact angle of T4 deposited in Ar plasma.

### 3.4. Surface Morphology

**Figure 5:**
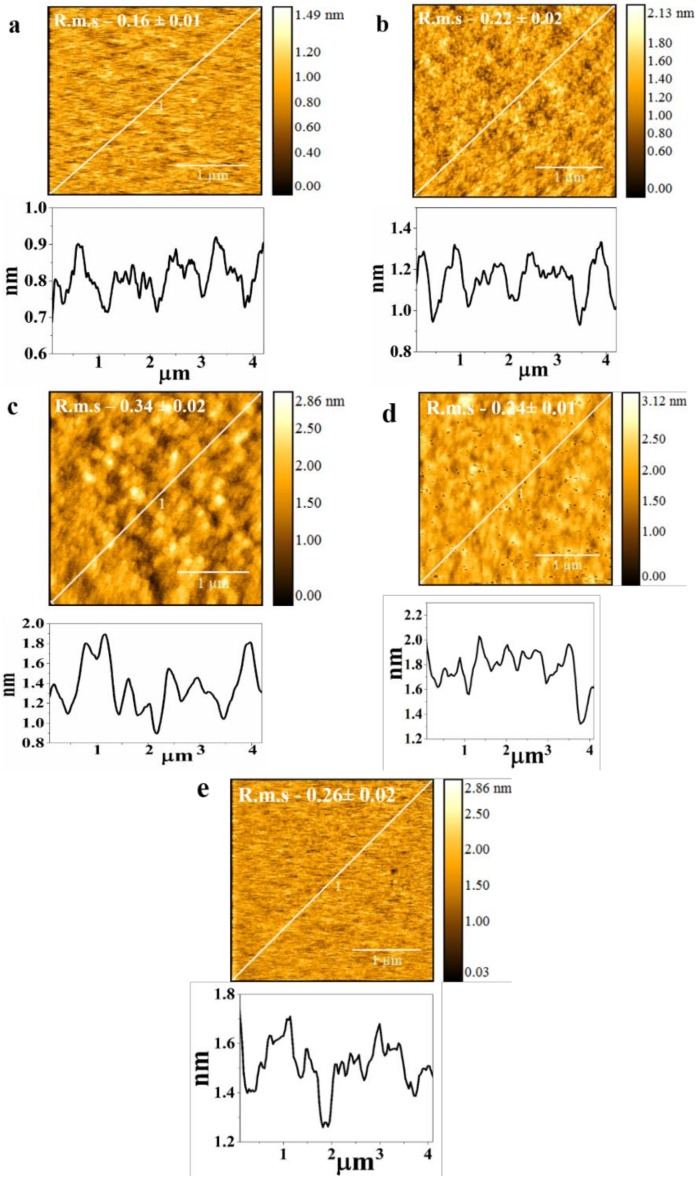
AFM images of (**a**) T4 _O2 plasma_, R.m.s = 0.16 ± 0.01 nm; (**b**) T4 _Ar_ _plasma_, R.m.s = 0.16 ± 0.02 nm; (**c**) T4 + ZnO, R.m.s = 0.34 ± 0.02 nm; (**d**) T4, R.m.s = 0.24 ± 0.01; (**e**) Silicon substrate, R.m.s = 0.26 ± 0.02. Scanning area 3 μm × 3 μm. Line profiles of surfaces are also shown.

**Figure 6:**
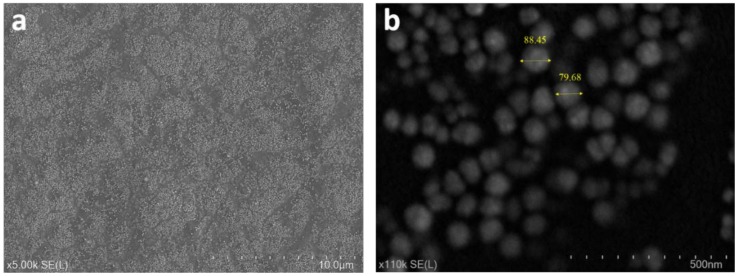
SEM images of ZnO modified T4 plasma polymers at different magnification of, (**a**) ×5K and (**b**) ×110K.

The effect of wettability and nanoparticle modification on surface roughness is shown in Figure 5. The root mean square (R.m.s) roughness of the oxygen and argon plasma deposited and ZnO modified T4 are 0.16 nm, 0.22 nm and 0.34 nm, respectively. The roughness of T4 without any treatment and of bare silicon substrate are 0.24 and 0.26 nm, respectively. The roughness reported here shows a nanometer scale. The ZnO-modified and Ar deposited T4 films have been found to show a cauliflower structure. However, this cauliflower morphology was not seen in samples deposited under oxygen plasma. The Ar deposited T4 exhibits a slightly increased roughness than that of the oxygen deposited samples. Figure 6 shows the SEM images of ZnO modified T4 films taken at various magnifications. The size of the nanoparticles ranged from 60 to 90 nm. Ball like ZnO nanoparticles were created according to growth conditions.

### 3.5. Light Transmission Efficiency

**Figure 7:**
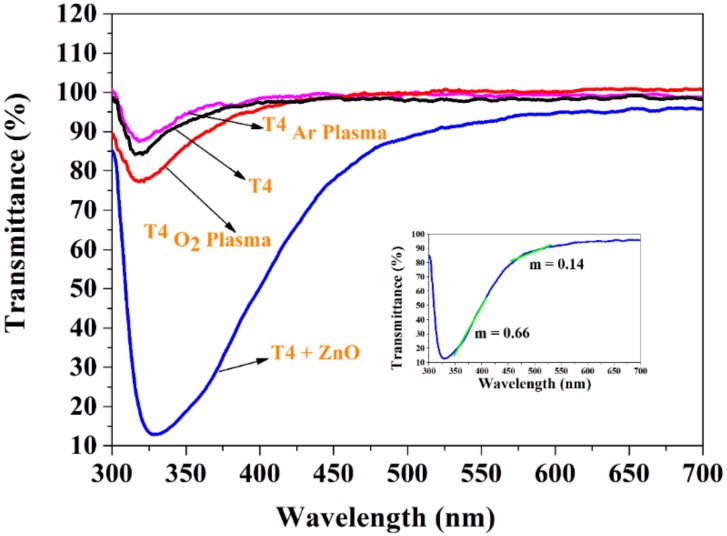
UV-visible spectra of modified T4 films. Inset graph shows the rate of decrease of UV and visible light transmission in two different regions between 300–400 nm and 450–600 nm.

Transparency to visible light and UV absorption of modified T4 film were measured within 200–1000 nm range. The transmittance spectra of the modified films are shown in Figure 7. T4 films functionalized with ZnO nanoparticles showed significant UV-blocking in the 300–400 nm region. UV absorption by ZnO nanoparticles seems to be a possible explanation for this phenomenon. It is also worthwhile to note that the rate of decrease in UV light transmission is higher than that of visible light. This rate of decrease is shown in the inset graph, where *m* denotes the slope of the graph in the particular region as marked by the green line. Thus, the UV blocking property of this film can be further enhanced by increasing the ZnO concentration on the film surface. Thin films with high UV absorption and visible light transmission have a potential application as optically transparent food packaging materials and there are many other applications [61,62]. Light transmission in the visible range of 400–700 nm is greater than 90% for T4 films deposited in argon and oxygen plasmas. The oxygen plasma-deposited T4 exhibited some UV blocking property, though it was significantly lower compared to ZnO-functionalized T4 films.

### 3.6. Thermal Degradation Behaviour

**Figure 8:**
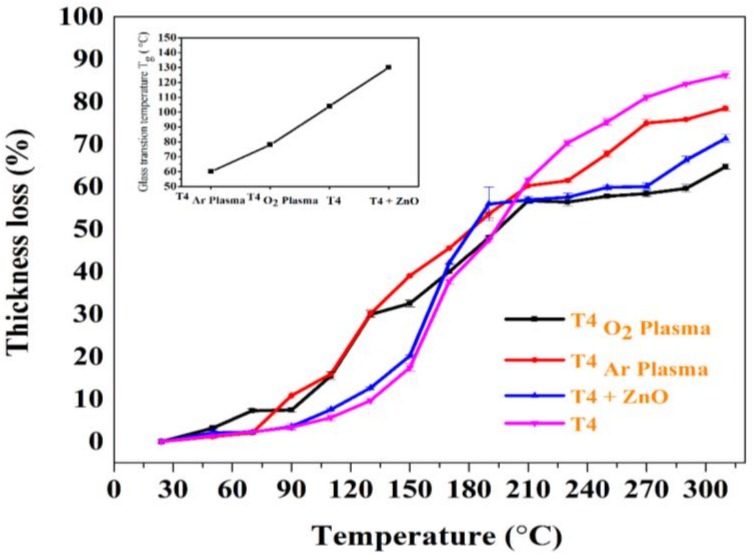
Percentage thickness loss as a function of temperature estimated using ellipsometry data. The inset shows the glass transition temperature T_g_ of modified T4 plasma polymers. T_g_ was found to increase in the following manner T4 _Ar plasma_ < T4 _O2 plasma_ < T4 < T4 + ZnO.

Thermal stability of modified T4 films was studied using in-situ dynamic ellipsometry [63]. A linear fit to acquired data over two temperature ranges was performed using the least square fit method. The glass transition temperature (T_g_) was determined by the intersection of two fitted lines. Figure 8 shows the percentage thickness loss as a function of temperature. Three kinds of thermal degradation regimes were observed for modified T4 films. Thermal degradation profile of the pristine T4 is also included for comparison. In the temperature regime of 50–90 °C, the rate of thickness loss was well below 20% for all the deposited films. Loss of trapped moisture and unbound species from the surface may explain this observation. Major thickness loss is observed in the temperature regime of 90–160 °C. This observation can be associated with cleavage of hydroxyl groups, decarboxylation reactions, and breaking of aliphatic carbon chains. The degradation phenomena above 170 °C can be attributed to breaking of carbon–carbon linkages. The inset in the graph shows the estimated T_g_ of modified T4 plasma polymers. The T_g_ of the modified T4 plasma polymer was found to increase in the following order, T4 _Ar plasma_ < T4 _O2 plasma_ < T4 < T4 + ZnO. This trend in T_g_ gives an insight into the chain length in the deposited polymers. The chain length is expected to follow the similar trend as T_g_. During deposition, higher T4 fragmentation occurs in argon plasma rather than in the oxygen plasma [48]. In addition, less T4 fragmentation is found when depositing without any carrier gases as evident from the trend above.

### 3.7. Antibacterial Test

**Figure 9:**
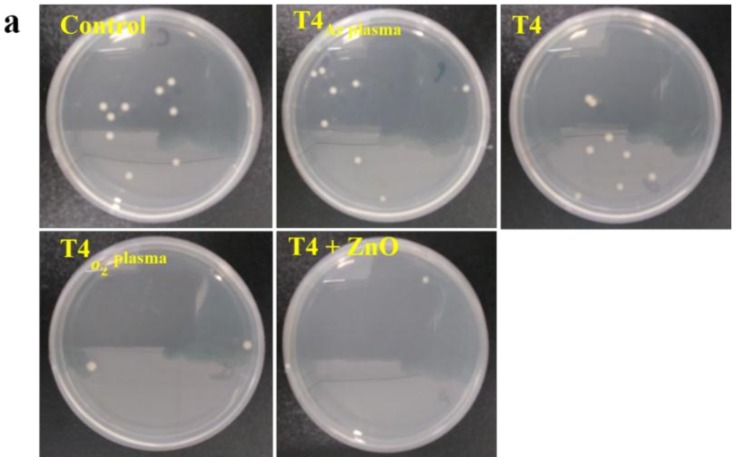

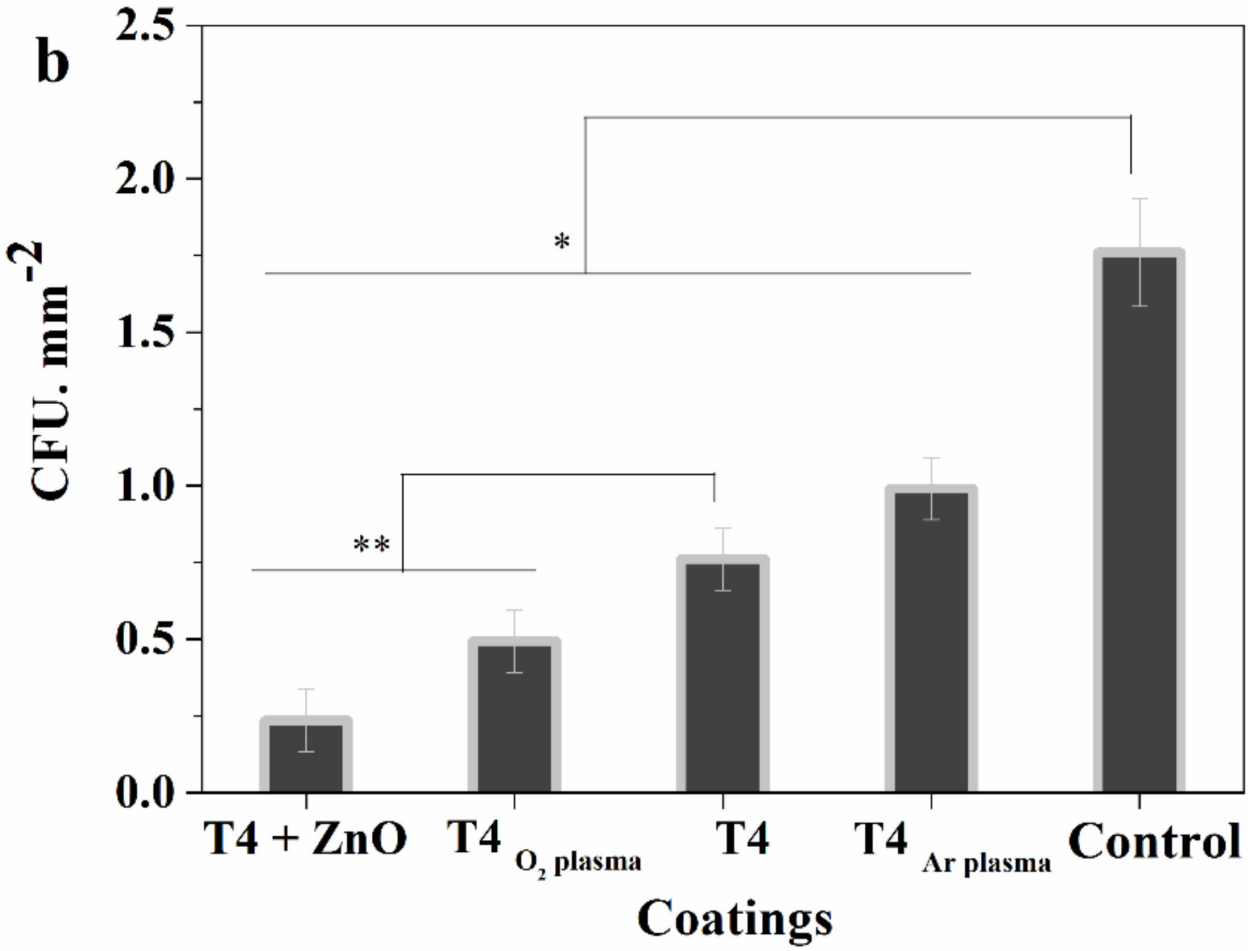
(**a**) photographs of *E. coli* colonies on agar culture plates after 24 h incubation on various T4 coatings; (**b**) colony forming units/mm^2^ on modified T4 surfaces and the control. All T4 surfaces showed a significant reduction in bacterial adhesion w.r.t. control (* *p* < 0.05). T4 + ZnO and T4 _O2 plasma_ demonstrated a significant reduction in bacterial adhesion w.r.t. to T4 (** *p* < 0.05).

Antibacterial activity of modified terpinen-4-ol surfaces was tested against *E. coli* cells. Initial adhesion up to 24 h was examined as it is the preliminary step in biofilm formation. Figure 9a shows the bacteria that survived after 24 h exposure to modified T4 surfaces and control. The small white dots are representative of *E. coli* colonies on agar culture plates. Figure 9b shows the colony forming unit (C.F.U.)/mm^2^ for all the modified T4 surfaces. All the modified T4 plasma polymers showed significant reduction in bacterial adhesion w.r.t control (*p* < 0.05). It can be observed that the T4 + ZnO and hydrophilic T4_O2 plasma_ surface exhibited a significant improvement in antibacterial activity w.r.t. pristine T4 (*p* < 0.05). The ZnO modified T4 demonstrated stronger antibacterial activity than T4_O2 plasma_, T4_Ar plasma_ and T4 films. In addition, T4_Ar plasma_ which is slightly hydrophobic showed more bacterial adhesion when compared to T4.

The combined effect of the hydrophilic surface along with the antimicrobial properties of T4 and ZnO is the possible reason for enhanced antibacterial performance of the T4_O2 plasma_ and T4 + ZnO polymers. Microorganism approaching the surface would find it difficult to settle on the surface due to the hydration layer formed between the surface and adjacent water molecules in the first place. This would be followed by the killing of any attached microorganism by ZnO nanoparticles and T4 molecules. The internalization of ZnO nanoparticles by cells is found to induce oxidative stress, resulting in their death [64,65]. In addition, T4 molecules have the ability to compromise the cell membrane structure, inhibiting their growth resulting in the cell death [66,67].

## 4. Conclusions

The use of argon and oxygen as a carrier gas had a profound influence on the deposition rate and degree of fragmentation of terpinen-4-ol during PECVD. Deposition in argon plasma leads to enhanced monomer fragmentation as compared to deposition occurring in oxygen plasma or in the absence of any carrier gas. This is also evidenced by the lowest T_g_, which is observed for T4_Ar plasma_. Chemical composition of terpinen-4-ol films deposited using either argon or oxygen as a carrier gas was found to be similar but varied in their relative peak magnitude. The ratio of −CH_x_/-OH group was greater in T4 films deposited in argon plasma than that in oxygen plasma. The difference in the proportion of functional group influenced the wettability of T4 films. Films rich in −OH content showed higher hydrophilic character. The oxygen plasma-modified and ZnO-modified T4 plasma polymers demonstrated enhanced antibacterial activity against *E. coli*. ZnO-modified T4 showed the most effective antibacterial activity among all the modified T4 coatings. Highly hydrophilic character combined with the antimicrobial effect of T4 and ZnO seems to be the possible explanation for this observation. Meanwhile ZnO-modified T4 coatings also showed significant UV absorption. However, as compared to unmodified T4 coatings, hydrophilic and ZnO nanoparticle modification played an important role in the enhancement of antimicrobial activity of T4 plasma polymers.

## Figures and Tables

**Table 1:** Contact angle of water, ethanediaol, and di-iodomethane on modified T4 surfaces.

Coatings	Contact Angle, °		
	Water	Ethane Diol	Di-iodomethane
**T4 + ZnO**	14.28 ± 0.58	15.96± 1.93	40.75 ± 0.73
**T4, *O_2_***	40.83 ± 0.36	16.63 ± 1.11	39.52 ± 0.77
**T4, *Ar***	75.19 ± 1.18	18.93 ± 0.57	37.85 ± 0.37
**T4**	65± 2.58	17.85 ± 3.45	37.83 ± 2.65
